# Association of Vitamin D and Prostate Health Status in Men: An Analytical Cross-Sectional Study

**DOI:** 10.7759/cureus.74959

**Published:** 2024-12-02

**Authors:** Janvitha Reddy, Arul Senghor K A, Vinodhini V M, N Prasath, Mansi Ravat

**Affiliations:** 1 Biochemistry, Sri Ramaswamy Memorial Medical College Hospital and Research Centre, Sri Ramaswamy Memorial Institute of Science and Technology, Kattankulathur, IND; 2 Medical Biochemistry, Sri Ramaswamy Memorial Medical College Hospital and Research Centre, Sri Ramaswamy Memorial Institute of Science and Technology, Kattankulathur, IND; 3 General Medicine, Sri Ramaswamy Memorial Medical College Hospital and Research Centre, Sri Ramaswamy Memorial Institute of Science and Technology, Kattankulathur, IND

**Keywords:** benign prostate hyperplasia, luts, prostate health, total psa, vitamin d

## Abstract

Introduction: Benign prostatic hyperplasia (BPH) is the most common form of lower urinary tract symptoms (LUTS). Vitamin D may be an effective way to treat BPH symptoms because it has anti-proliferative and anti-inflammatory characteristics. Thus, adequate vitamin D levels are vital for overall health. This study aimed to investigate the association of vitamin D with prostate health status in men and determine the diagnostic cut-off of vitamin D levels.

Methods: This analytical cross-sectional study was conducted among men between 45 and 80 years of age who presented to the surgery or urology department with complaints of frequent urination and urinary tract infection. Based on their total prostate-specific antigen (PSA) levels, the participants were categorized into four groups - Group A (total PSA 0-3.9 ng/ml), Group B (total PSA 4-9.9 ng/ml), Group C (total PSA 10 - 19.9 ng/ml), and Group D (total PSA > 20 ng/ml). Total PSA was estimated by a dedicated Beckman Coulter hormone analyzer (UNICEL DXI 600, Beckman Coulter, Inc., California, US) via the chemiluminescence method. Serum vitamin D was estimated with dedicated reagents in the Bio-Rad ELISA (Bio-Rad Laboratories, Inc., California, US). An analysis of variance (ANOVA) post hoc test was utilized to compare quantitative variables. A correlation analysis proved the relation between vitamin D and total PSA. The receiver operating characteristic (ROC) curve was utilized to determine the diagnostic performance of the analyte of interest.

Results: In this study, total PSA levels significantly increased in men over 75 years of age. The upper limit of total PSA increased with the age of the men. Total PSA levels were compared between the age groups of 40 to 49, 50 to 59, 60 to 69, and more than 70, and an increase in total PSA levels was observed in those between 60 and 69 years. Total PSA levels were also negatively correlated (r=- 0.31) with vitamin D in this age group. Moreover, vitamin D-deficient individuals had significantly increased total PSA levels (12.57±5.22 ng/ml). Further correlation analysis revealed a significant negative correlation of total PSA with vitamin D levels (r=- 0.245; p=0.022). ROC analysis had an area under the curve of 0.828 for Vitamin D at a cut-off of 20 ng/ml revealing a diagnostic sensitivity of 86% and specificity of 60%.

Conclusion: Vitamin D deficiency is a potential biochemical marker of benign prostate hypertrophy. Vitamin D and total PSA are indicators of prostate health status in men. Screening of total PSA levels reflects the prostate health status of men and this needs to be potentiated with vitamin D supplementation.

## Introduction

Men with benign prostate hyperplasia (BPH) usually present with frequent complaints of lower urinary tract symptoms (LUTS). BPH is characterized by stromal and epithelial cell proliferation in the prostate. Currently, 50% of males over 50 years of age and 90% over 80 years of age have prostate growth-related urinary symptoms [1,2]. Men with urological symptoms were found to be vitamin D deficient. This leads to a future therapeutic strategy for better health. LUTS in older men are generally connected to BPH and the latter is predicted to affect half of all males over the age of 50. Recently, an association between vitamin D insufficiency and LUTS in elderly men has been revealed [3].

Researchers conducted a dose-response meta-analysis and suggested that higher circulating vitamin D levels in patients with prostate cancer had reduced mortality. Thus, vitamin D was considered as a protective factor in the progression of prostate cancer [4]. Basic research has documented that the active form of vitamin D has cancer-fighting effects, where it binds to tissues and impacts the pro-differentiation and proliferation of tumor cells. In the prostate, vitamin D binds to specific receptors to inhibit prostate growth and inflammation. Research has demonstrated that vitamin D inhibits the pathological pathway in prostate stromal cells. This leads to the expression of cyclooxygenase 2 and favors the production of prostaglandin E2 (PGE2) in the prostatic stromal cells Researchers demonstrated the mechanism of action of vitamin D by its inhibition of the Rho-associated coiled-coil containing kinase (ROCK) pathway, leading to expression of cyclooxygenase 2 and production of PGE2 of prostate stromal cells [5].

Men diagnosed with BPH have increased inflammation that aggravates and worsens the pathogenesis. Proinflammatory cytokines stimulate the human prostate stromal cells, resulting in the production of IL-8, a chemokine that is involved in BPH pathogenesis. An analysis of studies summarized the role of vitamin D as having a role in spermatogenesis and prostate health. There is mechanistic evidence of anti-proliferative and anti-inflammatory properties of vitamin D in BPH. There is mechanistic evidence of anti-proliferative and anti-inflammatory properties of vitamin D in BPH [6]. In fact, vitamin D receptor agonists firmly suppress IL-8 production in BPH via the inhibition of the RhoA pathway, thereby decreasing prostate cell proliferation. According to a randomized controlled trial, vitamin D supplementation of 6000 IU per day in individuals with BPH significantly reduced the mean prostate volume [7]. Vitamin D supplementation is an effective way to treat BPH symptoms because it has anti-proliferative and anti-inflammatory characteristics [8]. The present study investigates the association of vitamin D and prostate health status in men and aims to determine the diagnostic cut-off for vitamin D levels.

## Materials and methods

This analytical cross-sectional study was conducted for six months at SRM Medical College Hospital and Research Centre, Kattankulathur, a tertiary health care center. Participants between 45 and 80 years of age were recruited by selective sampling. Institutional ethical committee approval was obtained from SRM Medical College Hospital and Research Centre (ethical clearance number: SRMIEC-ST0723-561). The sample size was calculated based on the prevalence of benign prostate hypertrophy in men [6].

Sample size (N) = Z2 (pq)/d2 where Z (level of confidence according to the standard normal distribution)=1.96; p (estimated proportion of the population)=7 %=0.07; q=1-p=1-0.07=0.93; d (tolerated margin of error); N=25.

The study was conducted among 88 men with complaints of frequent urination and urinary tract infection who presented to the surgery or urology department. Based on the total prostate-specific antigen (PSA) levels, the participants were categorized into Group A (total PSA 0-3.9 ng/ml), Group B (total PSA 4-9.9 ng/ml, Group C (total PSA 10-19.9 ng/ml), and Group D (total PSA>20 ng/ml). Individuals with major pelvic surgical interventions, abnormal digital rectal examination results, and previous total or transurethral prostatectomy were excluded.

The study was conducted from October 2023 to May 2024 in accordance with the Declaration of Helsinki. Protocols followed were as per the guidelines of biomedical research in human participants. The patient information sheet had the details, purpose, and procedure of the study. The detailed procedure was explained and written consent was obtained from every participant. After a physical examination by the medical officer, laboratory investigations were ordered. About 4 ml of venous blood samples were collected from the participants and were subjected to centrifugation after they clotted. The serum samples were analyzed to quantify the total PSA and vitamin D by chemiluminescence method in the hormone analyzer Beckman Coulter DXI 600 (Beckman Coulter, Inc., California, US). : The serum samples were stored in 2 ml Eppendorf tubes and kept at -20 degrees Celsius. These samples were analyzed for Vitamin D by ELISA technique in Bio-Rad reader (Bio-Rad Laboratories, Inc., California, US).

Statistical analysis

Data was analyzed using IBM SPSS Statistics for Windows, Version 22 (Released 2013; IBM Corp., Armonk, New York, United States) and used to compare quantitative variables. ANOVA was used to analyze the difference between the mean levels of the parameters between the two groups and P<0.05 was considered as statistically significant. Correlation analysis proved the relation between vitamin D and total PSA. The receiver operating characteristic (ROC) curve was utilized to determine the diagnostic performance of the analyte of interest. 

## Results

The participants were men between 40 and 80 years of age and were categorized into different age groups. Table 1 shows the comparison of total PSA (normal reference range 0-4.0 ng/ml) and vitamin D in the various age groups. For vitamin D, normal values are 30-100 ng/ml, where levels between 20 and <30 ng/ml are considered insufficient. Vitamin D deficiency is denoted by levels <20 mg/ml. 

**Table 1:** Comparison of total PSA and vitamin D levels in men based on their ages PSA: prostate-specific antigen ANOVA post hoc test: Data are expressed as mean and standard deviation, **Moderately significant

Parameters	40 – 49 years (n=18)	50 – 59 years (n = 23)	60 – 69 years (n = 29)	> 70 years (n=18)	p value
Vitamin D (ng/ml)	47.64 ± 3.32	55.65 ± 4.73	64.87 ± 4.55	76.56 ± 3.68	< 0.001**
Total PSA (ng/ml)	1.16 ± 0.36	11.58± 3.08	38.17 ± 13.78	26.81 ± 2.46	< 0.001**

**Figure 1:**
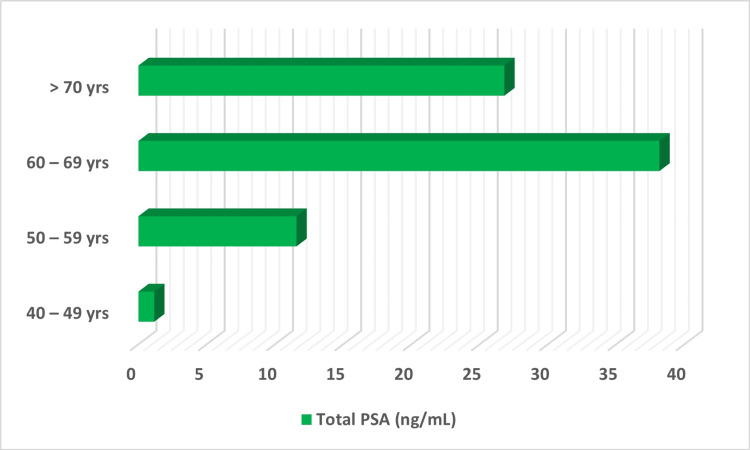
Total PSA levels based on age groups PSA: Prostate specific antigen

About 65% of the participants had total PSA levels within normal reference intervals, whereas 21.5%, 5.6%, and 8% of them had total PSA levels between 4 and 9.9 ng/ml, 10 and 19.9 ng/ml, and more than 20 ng/ml, respectively. Total PSA levels were elevated in those between 60 and 69 years of age compared to those less than 60 years old (Figure 1).

When vitamin D levels were compared between the four groups based on total PSA levels, the mean vitamin D levels were found to be normal in Group A which had normal total PSA levels. Group B had slightly higher total PSA levels and vitamin D levels were found to be slightly lowered in this group compared to Group A. Group C participants had elevated total PSA levels and insufficient vitamin D levels. Lastly, mean vitamin D levels were found to be deficient in Group D with total PSA >20 ng/ml. 

**Table 2:** Comparison of vitamin D levels based on levels of total PSA PSA: prostate-specific antigen ANOVA post hoc test: Data are expressed as mean and standard deviation, **Moderately significant, ***Highly significant

Parameters	Group A (Total PSA 0–3.9 ng/ml)	Group B (Total PSA 4–9.9 ng/ml)	Group C (Total PSA 10–19.9 ng/ml)	Group D (Total PSA >20 ng/ml)	p value
Total PSA (ng/mL)	1.3 ± 0.95	6.23 ± 2.02	16.45 ± 3.34	38.34 ± 15.3	< 0.001***
Vitamin D (ng/ml)	34.65 ± 8.53	31.98 ± 13.3	24.01 ± 9.2	18.31 ± 6.1	< 0.001**

**Figure 2:**
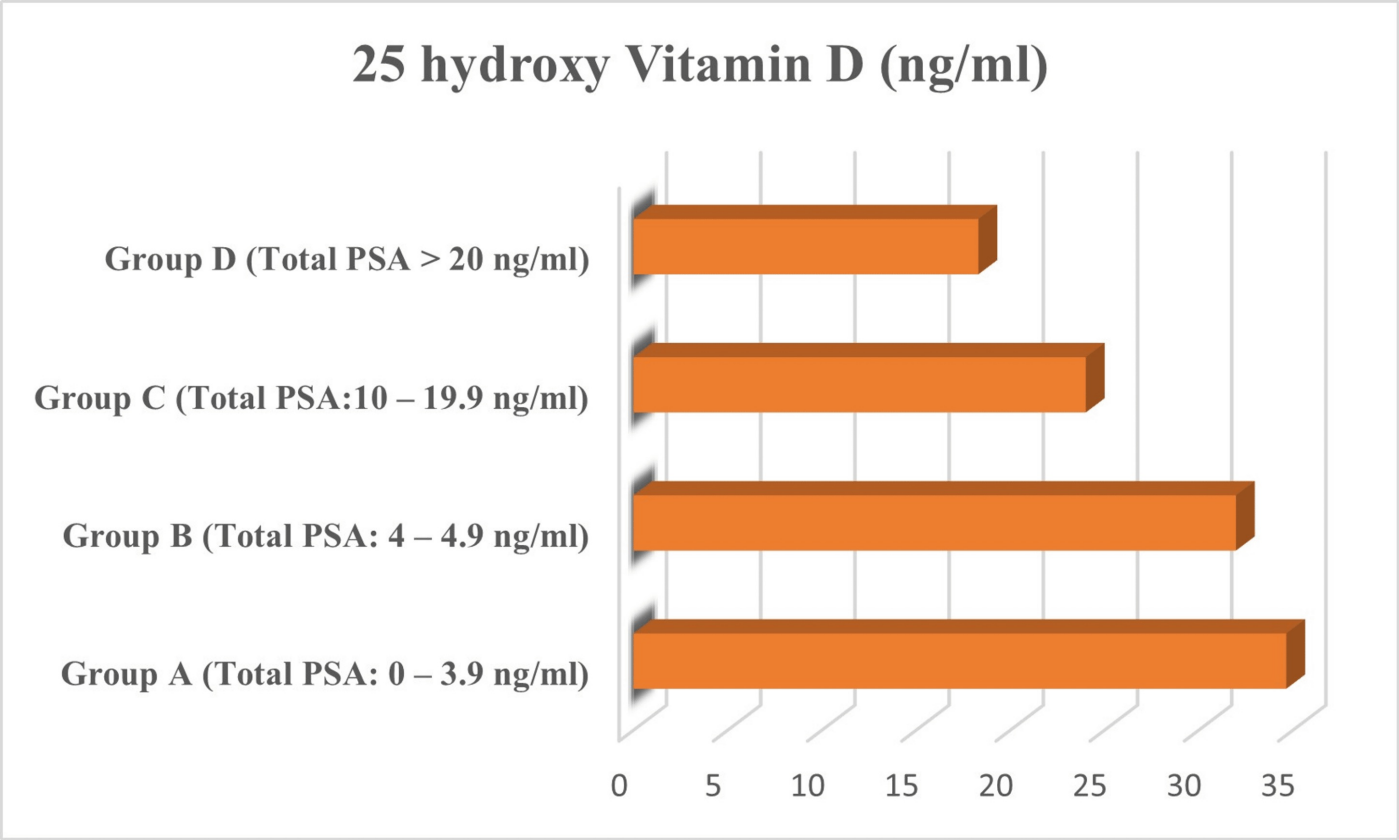
25-hydroxy vitamin D levels based on total PSA category PSA: prostate specific antigen

Vitamin D was decreased in the individuals with elevated total PSA levels (Figure 2). Further correlation analysis revealed a negative correlation of total PSA with vitamin D levels with an r value of -0.245 and a statistically significant p-value of 0.022.

**Figure 3:**
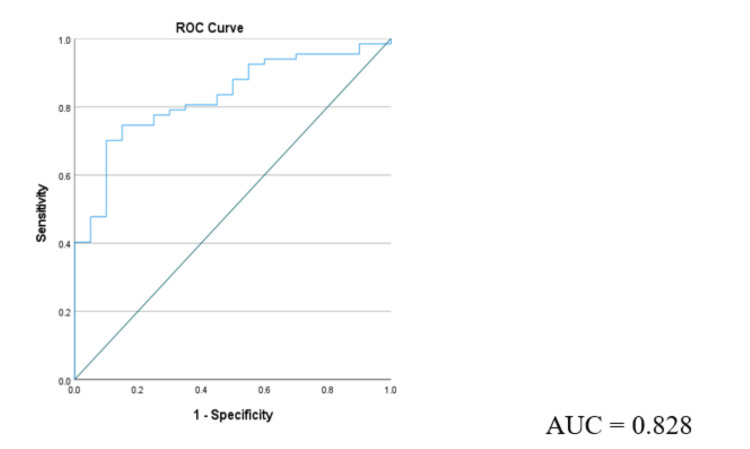
Receiver operating characteristic curve of 25 hydroxy vitamin D AUC: area under the curve, AUC is 0.828 for Vitamin D at a cut-off of 20 ng/ml

Figure 3 represents the ROC analysis of vitamin D in individuals with good and poor prostate health state based on the total PSA levels.

The area under the curve (Table 3) was found to 0.828 for Vitamin D at a cut-off of 20 ng/ml and revealed a diagnostic sensitivity of 86% and specificity of 60%.

**Table 3:** Receiver operating characteristics curve for vitamin D and prostate health Area under curve at 95% confidence interval, ***0.8-0.9: considered excellent.

Parameter	Cut-off value	Sensitivity (%)	Specificity (%)	AUC	Significance
25-hydroxy vitamin D (ng/ml)	19.9	86 %	60 %	0.828***	<0.000

## Discussion

Vitamin D plays a pivotal role in calcium homeostasis and potentially promotes health outcomes, via cancer prevention and modulation of immune function. Mandatory screening protocol for men focuses on total PSA which is a crucial biochemical marker that reflects men’s prostate health. It has been hypothesized that a vitamin D-deficient state is associated with high circulating total PSA levels [9,10]. The current study involves the identification of men with vitamin D-deficient states and the analysis of total PSA. This can help in the development of new therapeutic strategies to promote healthy prostate health in the aging male population.

Master health checkups for men had a routine panel of investigations that included total PSA and vitamin D. There was an increase in total PSA values in the male population between 60 and 69 years. The most common genitourinary diseases in men include BPH and prostate cancer. As BPH prevalence increases with age, it is essential to consider potential preventive and therapeutic measures. Recent research has explored the role of vitamin D in BPH, given its widespread biological effects, including regulation of cell growth, differentiation, and immune function. Also, therapeutic intervention with vitamin D can reduce overtreatment of non-aggressive prostate cancer.

Gao et al. implemented a risk stratification model with a free/total PSA ratio in a retrospective study with 1807 participants [11]. In this study, a comparative analysis of vitamin D was conducted in those with an increased frequency of urination and urinary tract symptoms. It was observed that 18.2% of patients with increased total PSA levels had inadequate vitamin D levels.

In 2020, a randomized controlled trial was conducted to investigate the impact of vitamin D supplementation on the advancement of BPH [7]. In the study, those who received vitamin D had considerably lower PSA levels than the control group. The group given vitamin D supplementation also showed a drop in the International Prostate Symptom Score, a measure of BPH severity. This could be due to the beneficial role of vitamin D in reducing inflammation and cell proliferation associated with BPH. Calcitriol supplementation inhibits prostatic stromal and epithelial cell proliferation, induces apoptosis, and reduces prostatic enlargement. Moreover, it can inhibit the fibrotic process, characteristic of structural changes in BPH.

One population-based study hypothesized the epidemiological relationship between vitamin D levels and risk of prostate cancer incidence in 2578 middle-aged men. During follow-up, 296 men had prostate cancer, especially in the group with vitamin D deficiency [12]. In this study, the increase in total PSA with inadequate vitamin D levels was predominant, especially in the age group of 60 to 69 years. Crosstalk between the vitamin D and androgen receptor signaling pathways explains the key pathway that directs prostate pathology. Androgen receptors are expressed in androgen-dependent tissues such as the prostate. Thus, androgen deprivation is the ideal treatment strategy for patients with advanced and metastatic prostate cancer [13]. Vitamin D receptors, on the other hand, are expressed in Leydig cells, Sertoli cells, and seminiferous tubules and are involved in the androgen signaling mechanism. Vitamin D has been suggested as the protective factor that maintains prostate health.

The significant inverse correlation of total PSA with vitamin D levels adds to the picture of the negative impact of a deficient state on prostate health in men [14]. Similarly, Thederan et al. had reported low circulating levels of vitamin D and selenium in prostate cancer patients with a negative correlation between vitamin D and total PSA levels [15]. A prospective case-control study conducted in 156 advanced prostate cancer patients measured vitamin D and vitamin D derived binding protein (VDBP), and proved the association of VDBP and bioavailable vitamin D with lethal prostate cancer [16]. Growth differentiation factor (GDF)-15 also known as macrophage inhibitory cytokine-1, is an anti-inflammatory signalling molecule. GDF-15 was found to have inverse association with prostatic inflammation. Also, vitamin D regulates many signaling pathways that relieve inflammation, protect prostatic cells from inflammatory cytokines, and alleviate their oxidative stress [17,18]. Our study showed that men with BPH present with increased total PSA and decreased circulating vitamin D levels. Moreover, a diagnostic sensitivity of 86% and specificity of 60% was demonstrated for vitamin D at a cut-off value of 20 ng/mL.

The limitations of the study include a small sample size due to which the results cannot be generalized for the entire male population. Moreover, the study design is a cross-sectional study which precludes long-term effects. Longitudinal studies with vitamin D supplementation and follow-up would provide more comprehensive insights. Researchers can potentially uncover the trends and molecular mechanisms that strengthen the interlinking between vitamin D and prostate health in men.

## Conclusions

The study concluded that vitamin D deficiency was negatively correlated with total PSA levels in individuals with BPH. Vitamin D and total PSA are indicators of prostate health status in men. A total PSA screening tool can reflect the prostate health status of men and help identify the need for vitamin D supplementation, which can be implemented at the earliest. Hence, a routine biochemistry panel for screening prostate health in men should consider total PSA and vitamin D levels so that individuals deficient in vitamin D are identified and interventional strategies can be planned for their well-being.
